# Reliability of Frail and Barthel Tests for Detecting Frailty in Palliative Oncological Patients in a Home Hospitalization Unit: A Comparative Study

**DOI:** 10.3390/life12020286

**Published:** 2022-02-14

**Authors:** Susana León-Ramón, Emmanuel Navarro-Flores, Marta Elena Losa-Iglesias, Ricardo Becerro-de-Bengoa-Vallejo, Ana María Jiménez-Cebrián, Carlos Romero-Morales, Patricia Palomo-López, Daniel López-López

**Affiliations:** 1 Home Hospitalization Unit, General University Hospital of Valencia, Department of Nursing, Faculty of Nursing and Podiatry, University of Valencia, 46001 Valencia, Spain; sulera@alumni.uv.es; 2 Frailty Research Organized Group, Department of Nursing, Faculty of Nursing and Podiatry, University of Valencia, 46001 Valencia, Spain; 3 Faculty of Health Sciences, Universidad Rey Juan Carlos, 28922 Alcorcón, Spain; marta.losa@urjc.es; 4 School of Nursing, Physiotherapy and Podiatry, Universidad Complutense de Madrid, 28001 Madrid, Spain; ribebeva@ucm.es; 5 Department Nursing and Podiatry, Faculty of Health Sciences, Instituto de Investigación Biomédica de Málaga (IBIMA), University of Malaga, 29010 Malaga, Spain; amjimenezc@uma.es; 6 Faculty of Sport Sciences, Universidad Europea de Madrid, Villaviciosa de Odón, 28001 Madrid, Spain; carlos.romero@universidadeuropea.es; 7 University Center of Plasencia, Universidad de Extremadura, 10600 Plasencia, Spain; patibiom@unex.es; 8 Research, Health and Podiatry Group, Department of Health Sciences, Faculty of Nursing and Podiatry, Universidade da Coruña, 15403 Ferrol, Spain; daniellopez@udc.es

**Keywords:** frailty, palliative care, cancer, home care, frail-VIG index

## Abstract

Cancer is a condition that can increase the risk of frailty. In addition, palliative oncological patients in home hospitalization can find their activities of daily living affected. The main objective was to measure the degree of frailty in the oncological population in home hospitalization comparing Barthel and Frail-VIG Indexes. This is a descriptive cross-sectional study. A sample of oncological patients in home hospitalization (*n* = 50) that included 27 men and 23 women were recruited, and disability due to frailty was measured using the VIG frailty index and the Barthel scale for Activities of Daily Living (ADLs). Spearman’s correlation coefficients were categorized as weak (rs ≤ 0.40), moderate (0.41 ≤ rs ≥ 0.69) or robust (0.70 ≤ rs ≥ 1.00), with a strong indirect correlation between the domains using the toilet, transferring and wandering on the Frail-VIG scale with an r (s) value −0.810 (*p* < 0.001), −0.831 (*p* < 0.001) and −0.805 (*p* < 0.001), respectively, and a moderate indirect correlation for the domains of eating −0.718 (*p* < 0.001), dressing −0.770 (*p* < 0.001) and urination −0.704 (<0.001). The Frail-VIG index above 0.35 points, that is, from moderate to severe, does not affect ADLs except in the nutritional dimension in a palliative oncological population in home hospitalization. The preliminary outcomes obtained should be considered to determine the impact of nutritional status with regard to ADLs in palliative oncological patients in a home hospitalization unit.

## 1. Introduction

There are many factors that have made cancer one of the main causes of death worldwide today; one of them is the increase in life expectancy since many cancers manifest in advanced age [1]. When cancer is incurable we have recourse to palliative care, which is defined by the WHO as that aimed at “improving the quality of life of patients and their families when faced with the physical, psychological, social or spiritual problems inherent in a life-threatening disease” [1,2,3]. That is why a large number of patients receiving palliative care are elderly since aging and cancer are related [1].

Cancer is one of the leading causes of death worldwide, amounting to approximately 9.9 million deaths in 2020 according to the International Center for Research on Cancer (IARC) and is expected to continue to increase [4].

In Spain, the number of diagnosed cancers is expected to reach 276,239 in 2021, according to calculations by the Spanish Network of Cancer Registries (REDECAN) [5,6]. Therefore, this indicates that, in many cases, palliative care will end up being one of the main resources [7].

When we refer to palliative care we should be aware that it is not restricted to the last years of life, for it must be proportional according to the needs of the patients and family during the development of the disease [8].

We can define frailty as a state of great vulnerability due to different stress factors that decrease endurance, strength and functional reserve, causing a greater risk of dependence and/or death [9,10,11].

There are several levels of frailty that can help us establish the situational diagnosis of a patient. These indices are used with older people, but frailty has not been studied in the field of palliative oncology with seniors; in fact, few tools are available for assessing the degree of frailty in this type of patient [12].

Based on deficits in the activities of daily living that the patients present, the results on frailty were measured with a continuous variable, the degree of vulnerability, and thus the biological age of the study subjects [13].

There is prior clinical research in the geriatric population and oncological process with regards frailty and activities of daily living [14,15].

In this observational study we are going to use the Frail-VIG Index (comprehensive geriatric assessment) [16], in order to analyze the degree of frailty of palliative cancer patients admitted to the Home Hospitalization Unit (HHU) at the General Hospital of Valencia, Valencia, Spain.

The HHU is an alternative to conventional hospitalization, and the patient is attended to by health professionals in the same manner as in the hospital but at home, with the aim of providing all the care and palliative treatments needed, both for the patient and for their family.

“Biologic validity refers to the closeness of scale assessments to the hypothesized expectation when comparing with other measures in a specific population” [17]. Thus, the null hypothesis of this study is that the frailty VIG test and Barthel test have the same reliability in palliative cancer patients admitted to the HHU. The frailty VIG test and Barthel tests have the same reliability in palliative cancer patients admitted to the HHU. We aimed to analyze the psychometric properties of the Frail-VIG index and the Barthel test in a sample of palliative cancer patients.

## 2. Materials and Methods

### 2.1. Design and Sample

This is a cross-sectional descriptive observational study, following the STROBE methodology to carry out observational studies [18], conducted during the months of October 2020 to May 2021 in the HHU service at the General University Hospital of Valencia, Spain; an acute care hospital with a capacity of 503 beds that covers a reference population of 364,000 inhabitants.

Fifty palliative cancer patients admitted to the HHU were included at the time of data collection, in order to analyze frailty using the Frail-VIG Index.

The determination of the sample size was calculated with G * Power 3.1.9.2 (Heinrich-Heine-Universität Düsseldorf; Düsseldorf, Germany) after testing the correlation between two paired means with respect to the correspondence with a Spearman correlation coefficient of 0.40 and a 95% confidence interval (CI) for a two-tailed test, with an α error of 0.05, an estimated analysis power of 80% (β error = 20%), estimating a final sample size of 46 participants [19].

The sample included patients aged between 41 and 96 years old diagnosed with a palliative oncological disease, both men and women, who were being treated by the HHU team. The exclusion criteria were all patients with a very poor prognosis, whose life expectancy was less than 15 days at the time of data collection. Furthermore, according to Mini-Mental State Examination (MMSE) cognitive function, patients with cognitive impairment, a score less than 27, were excluded.

### 2.2. Procedure

*A* palliative care nurse (SLR) performed a cognitive function assessment to establish the participant’s cognitive eligibility. This was performed according to Mini-Mental State Examination (MMSE) for analysis of cognitive function [20]. The MMSE is a quick and easy measure that assesses seven areas of cognitive functioning, and it was shown to have both good test-retest reliability (0.80–0.95) [21] and acceptable sensitivity and specificity to detect mild to moderate stages of dementia [22].

Following this evaluation, the investigators explained the study procedures in detail to the participant. The interviews comprised questions on general health status, sociodemographic characteristics (sex, age, body mass index, height and weight) and comorbidities (e.g., anxiety, depression, diabetes, obesity, osteoarticular diseases, vascular disorders or kidney illness). Data on comorbidities were collected from the patients’ medical records.

The Frail-VIG Index [23] the Frail-VIG index showed better inter-rater reliability (0.94), and the test–retest was also excellent (0.97), indicating that if frail elderly people are stable and the Frail-VIG index is administered under similar conditions, their scores remain stable over time.

The Frail-VIG Index was used to determine the degree of frailty of the study population and consists of 22 questions divided into eight domains, in which the following were evaluated: functional domain; divided between the IADL (instrumental activities of daily living): money management, use of the telephone, compound medication control, and on the other hand, ADLs (using the Barthel Index): the nutritional domain (evaluating weight loss ≥5% in the last 6 months); the cognitive domain; the emotional domain: depressive syndrome and insomnia/anxiety; the social domain (social vulnerability); geriatric syndromes: delirium, falls, ulcers, polypharmacy, dysphagia; severe symptoms: pain and dyspnea; and diseases: cancer and respiratory, cardiac, neurological, digestive and renal diseases [19].

The total scores determine four degrees of frailty: prefrailty, baseline, moderate, and advanced, with values of <0.20 points, 0.20–0.35 points, 0.36–0.50 points and >0.50 points, respectively [16].

The Barthel index is an instrument used in medicine for the functional assessment of a patient [24,25]. This scale is used to measure the ability of a person to perform 10 basic ADLs; in this way, a quantitative estimate of their degree of independence can be obtained regarding ADL in institutionalized nursing home patients, for example, nutrition or walking, with a score from 0 to 100 [26].

Once all of the scores are obtained, the sum is organized in such a way that the totally independent residents will have a score of 100, the residents with mild dependency will have a score of 91 to 99 points, moderate dependency is established with a score of 61 to 90, severe dependence entails scores ranging from 21 to 60 points, and total dependence is considered as a score of 20 points or less [27].

This index has been described by many authors as the most widely used index for evaluating ADL in chronically ill patients and periodically evaluating their progression [28,29,30]. The reliability of the test according to Cronbach’s alpha is 0.86–0.92 for the original version and 0.90–0.92 for the version proposed by Shah et al. [29].

### 2.3. Ethical Considerations

This study was approved by the Ethics Committee of the CHGUV code 171/2020. Patients or, failing that, primary caregivers who decided to participate voluntarily signed the patient information sheet and informed consent at the time of data collection.

### 2.4. Analysis of Data

All of the variables were normally distributed, as determined by the Kolmogorov–Smirnov test (*p* > 0.05). Parametric data were determined using the mean, standard deviation (SD), and minimum and maximum (range) values. A comparison of quantitative data was made for the different subscales of the Frail-VIG questionnaire and the Barthel scale, and significant differences were verified using Student’s t test for independent samples. Spearman correlation coefficients (rs) were determined and scored as low rs ≤ 0.40, moderate 0.41 ≤ rs ≥ 0.69 or robust 0.70 ≤ rs ≥ 1.00.

Furthermore, convergent validity was assessed by examining the correlation between Frail-VIG test and Barthel Scale total scores and each item. At the same time, the correlation values are considered to indicate good correlation when 0.41–0.60, signify very good correlation when 0.61–0.80, and signify excellent correlation when >0.81 [31].

Reliability was assessed through measurements of internal consistency, test–retest reliability, and interrater reliability. The internal consistency of the Frail VIG Scale and the Barthel Scale was measured using Cronbach’s α coefficient. The criterion of Cronbach’s alpha > 0.70 and the Intraclass Correlation Coefficients (ICC) > 0.70 were used to assess the internal reliability and test–retest reliability, respectively [32]. The value of ICC was calculated again after a 1 week interval.

The data were analyzed using Spearman’s correlation coefficients. Interrater reliability was calculated between two neuropsychologists using the intraclass correlation coefficient (ICC). To assess the concurrent validity between domains of the Frail VIG Scale and the Barthel Scale, Spearmen’s correlation coefficient was used to compare both tests.

All analyses were considered statistically significant when the *p*-value was <0.05 with a 95% confidence interval (CI). Statistical analyses were performed with SPSS (v26.0, Chicago, IL, USA).

## 3. Results

### 3.1. Descriptive Data and Sociodemographic Data

Significant differences were shown in age, height, weight and BMI (*p* < 0.05). The sample included 50 subjects, 23 women and 27 men, whose mean age was 79.78 ± 10.87 years. Table 1 shows the sociodemographic characteristics.

### 3.2. Psychometric Assessment

Results and systematic differences of the Frail-VIG index and Barthel score subscales are indicated in Table 2 and Table 3, respectively.

An adequate Cronbach’s alpha was indicated for some of the main domains of VIG in diseases (α = 0.730), geriatric syndromes (α = 0.765), and cognitive impairment (α = 0.724), as well as for the Barthel index eat (α = 0.933), wash (α = 0.935), and urination (α = 0.933).

The results of Table 4 show a strong indirect correlation between domains using the toilet, moving and wandering on the Frail-VIG scale with an r value (s) −0.810 (*p* < 0.001), −0.831 (*p* < 0.001) and −0.805 (*p* < 0.001), respectively, and a moderate indirect correlation for the domains eat −0.718 (*p* < 0.001), dress −0.770 (*p* < 0.001) and urination −0.704 (<0.001).

According to the results, there were 4 (8%) prefrail patients, 9 (18%) with slight frailty, 25 (50%) with moderate frailty and 12 (2.4%) with advanced frailty.

The Frail-VIG Index and Barthel Index did not show a normal distribution (*p* < 0.05). The Spearman correlation coefficient between the Frail-VIG Index and the Barthel Index shows a more robust correlation for nutritional status, with an r (s) value of 0.820 (*p* = 0.569).

Other results shown in Table 5, which include the items with higher percentages, are of malnutrition, polypharmacy and anxiety/depression [33,34].

Because this study is based on oncological palliative patients, the dimensions of frailty are clearly related to the problems most common to this type of patient because some of the more frequent symptoms in advanced cancer are: lack of appetite, nausea, vomiting, constipation, diarrhea, insomnia, anxiety, depression, dyspnea, and pain, among others [35].

## 4. Discussion

It is evident that most of the palliative oncological patients admitted to the HHU have high degrees of frailty and different levels of dependence for ADLs. Thus, malnutrition, polypharmacy and anxiety/insomnia are significant in this study, as they are disorders that appear at the end of the patient’s life [36].

In our study, 68% had a total weight loss of 5% during the previous 6 months, since cachexia is a common symptom of oncological diseases [37], characterized by loss of muscle mass and progressive functional impairment, which is impossible to reverse; similar results to those of the study by Amblás-Novellas et al. [38], which also focuses on palliative patients, with 63.2% of the study subjects affected by this symptom.

The objectives in our research were achieved, and the characteristics of these questionnaires make them ideal for clinical use. These scales should also be used as a complement to compound indices of disease activity such as PROMS [14,23]. In fact, our results were similar to the results of Torné [23]. In our research, the study subjects showed a Frail-VIG score to assess the degree of frailty in an oncological home hospitalization unit with a lower result. This can be considered as a gold standard according to the results obtained that supported the Frail-VIG index as a reliable, feasible, and valid tool to assess the degree of frailty.

On the other hand, other studies show more disparate results since they do not focus only on specific patients as such but are based on elderly patients in general. An example of this is the study by Mota-Romero et al. [34], where only 8.7% show malnutrition, or another study by Amblas-Novellas [16] with 32.2%. In contrast, all the aforementioned studies match ours in the percentage of poly medicated patients since 84% of our patients take more than five drugs, similar to the 83.2% in the study by Mota-Romero et al. [34] and 80% in that by Amblás-Novellas [15], as there is a close relationship between advanced age and polypharmacy for symptom management. Ninety-two percent of the patients had some type of frailty, a percentage very similar to those in the study by Amblás-Novellas et al. [16] in which 92.5% were frail palliative patients.

The mean was 79.78 years, which is lower than that of other studies carried out by Zamora-Sánchez et al. [39], or Amblás-Novellas et al. [15], where the mean ages were 88 and 86.4 years, respectively.

In our study, the Spearman indirect correlation coefficient between the Frail-VIG index and the Barthel index showed that the higher the Barthel index score, the lower the result in the Frail-VIG index. Several limitations of this research must be considered; for example, a population from different territories would be useful to improve results.

The results show that ADLs can reflect a greater degree of frailty in palliative oncological patients in a home hospitalization unit, and it has been found that ADLs are not affected in terms of frailty according to the results of the Frail-VIG index, except in the case of aspects related to the functional aspects of the Barthel index.

Although ambulation and functional performance and the risk of falling are very common in frail people [19,40], this research should also be developed in other population groups to determine the degree of frailty, for example, in widows who tend to have higher scores for frailty due to psychosocial aspects [40,41].

In addition, selective sampling can lead to biases; for this reason, random sampling should be considered in future studies.

Ultimately, the impact of the correlation between different dimensions of frailty, such as cognitive impairment, was not analyzed in our research because the population studied was not adequately adjusted to develop these comparisons. Therefore, the researchers suggest that future research should be carried out on patients with different cognitive disorders.

## 5. Conclusions

Over 0.35 points on the Frail-VIG index, i.e., moderate to severe, does not affect ADLs except in the nutritional dimension in a population of palliative oncological patients in home hospitalization. The preliminary outcomes obtained should be considered to determine the impact of nutritional status with regard to ADLs in palliative oncological patients in a home hospitalization unit.

## Figures and Tables

**Table 1:** Descriptive and sociodemographic data of the sample.

Demographic and Descriptive Data	Total Group *n* = 50 Mean ± SD (Rank)	Woman *n* = 23 Mean ± SD (Rank)	Man *n* = 27 Mean ± SD (Rank)	*p*-Value
**Age (years)**	79.78 ± 10.87 (76.68–82.87)	80.73 ± 1.37 (76.25–85.22)	78. 96 ± 11. 41 (74.44–83.47)	<0.001
**Weight (kg)**	64.54 ± 14.56 (60.39–68.68)	61.41 ± 14.70 (55.05–67.77)	67.20 ± 14.18 (61.59–72.81)	<0.001
**Height (m)**	1.62 ± 0.09 (1.60–1.65)	1.55 ± 0.06 (1.52–1.58)	1.68 ± 0.06 (1.65–1.71)	<0.001
**BMI (kg/m^2^)**	24.32 ± 4.92 (22.92–25.72)	25.21 ± 5.59 (55.05–67.77)	23.57 ± 4.24 (21.89–25.25)	<0.001

BMI: body mass index; In all analyses, *p* < 0.05 (with a 95% confidence interval) was considered statistically significant.

**Table 2:** Reliability results, item-total correlation on the Frail-VIG Index according to each item.

*n* = 50					
Frail-VIG Index		Mean ± SD (95% CI)	Item Correlation—Corrected Total	Cronbach’s Alpha If Element Is Deleted	*p*-Value
**FUNCTIONAL**	**Item 1. Money management**	0.64 ± 0.48 (0.50–0.77)	0.294	0.714	<0.001
	**Item 2. Using the telephone**	0.44 ± 0.50 (0.29–0.58)	0.630	0.688	<0.001
	**Item 3. Medication control**	0.52 ± 0.50 (0.37–0.66)	0.677	0.684	<0.001
	**Item 4. Barthel Index**	1.60 ± 0.94 (1.33–1.86)	0.755	0.650	<0.001
**NUTRITIONAL**	**Item 5. Malnutrition**	0.68 ± 0.47 (0.54–0.81)	0.134	0.632	<0.001
**COGNITIVE**	**Item 6. Degree of cognitive impairment**	0.48 ± 0.64 (0.29–0.66)	0.475	0.724	<0.001
**EMOTIONAL**	**Item 7. Depressive syndrome**	0.20 ± 0.40 (0.08–0.31)	0.028	0.696	<0.001
	**Item 8. Insomnia/Anxiety**	0.62 ± 0.49 (0.48–0.75)	0.350	0.730	<0.001
**SOCIAL**	**Item 9. Social vulnerability**	0. 10 ± 0.30 (0.01–0.18)	−0.025	0.710	0.024
**GERIATRIC** **SYNDROMES**	**Item 10. Delirium**	0.20 ± 0.40 (0.08–0.31)	0.285	0.730	<0.001
	**Item 11. Falls**	0.16 ± 0.37 (0.05–0.26)	−0.050	0.715	0.004
	**Item 12. Ulcers**	0.28 ± 0.45 (0.15–0.40)	0.380	0.733	<0.001
	**Item 13. Polypharmacy**	0.84 ± 0.37 (0.73–0.94)	0.301	0.708	<0.001
	**Item 14. Dysphagia**	0.14 ± 0.35 (0.04–0.23)	0.176	0.715	0.007
**SEVERE** **SYMPTOMS**	**Item 15. Pain**	0.64 ± 0.48 (0.50–0.77)	−0.069	0.721	<0.001
	**Item 16. Dyspnea**	0.26 ± 0.44 (0.13–0.38)	−0.003	0.738	<0.001
**DISEASES**	**Item 17. Cancer**	1.00 ± 0.00	NA	0.733	NA
	**Item 18. Respiratory**	0.30 ± 0.46 (0.16–0.43)	−0.233	0.747	<0.001
	**Item 19. Cardiac**	0.42 ± 0.49 (0.27–0.56)	0.185	0.721	<0.001
	**Item 20. Neurological**	0.14 ± 0.35 (0.04–0.23)	0.241	0.718	0.007
	**Item 21. Digestive**	0.34 ± 0.47 (0.20–0.47)	−0. 006	0.734	<0.001
	**Item 22. Renal**	0.32 ± 0.47 (0.18–0.45)	0.240	0.717	<0.001

CI: confidence interval; Student’s t tests were used. In all analyses, *p* < 0.05 (with a 95% confidence interval) was considered statistically significant.

**Table 3:** Reliability results, item-total correlation of the Barthel Index according to each item.

*n* = 50				
Barthel Index	Mean ± SD (95% CI)	Item Correlation—Corrected Total	Cronbach’s Alpha If Element Is Deleted	*p*-Value
**Eat**	7.20 ± 3.66 (6.15–8.24)	0.724	0.933	<0.001
**Wash**	2.40 ± 2.52 (1.68–3.11)	0.737	0.935	<0.001
**Dress**	4.70 ± 3.55 (3.68–5.71)	0.796	0.930	<0.001
**Get ready**	2.80 ± 2.50 (2.08–3.51)	0.724	0.936	<0.001
**Depositions**	6.30 ± 3.88 (5.19–7.40)	0.642	0.937	<0.001
**Urination**	5.30 ± 4.33 (4.06–6.53)	0.723	0.933	<0.001
**Toilet**	5.00 ± 3.91 (3.88–6.11)	0.843	0.928	<0.001
**Transfers**	8.70 ± 5.32 (7.18–10.21)	0.891	0.925	<0.001
**Ambulation**	9.00 ± 5.89 (7.32–10.67)	0.891	0.927	<0.001
**Steps**	3.40 ± 3.56 (2.38–4.41)	0.750	0.932	<0.001

CI: confidence interval; NA: Not applicable. Student’s t tests were used. In all analyses, *p* < 0.05 (with a 95% confidence interval) was considered statistically significant.

**Table 4:** Spearman correlations between domains of the VIG Scale and the Barthel Scale.

Frail-VIG Index	Barthel Eat r (P)	Barthel Wash r (P)	Barthel Dress r (P)	Barthel Get Ready r (P)	Barthel Depositions r (P)	Barthel Urination r (P)	Barthel Use the Toilet r (P)	Barthel Move r (P)	Barthel Wander r (P)	Barthel Steps r (P)
**FUNCTIONAL**	−0.718 (<0.001)	−0.650 (<0.001)	−0.770 (<0.001)	−0.661 (<0.001)	−0.637 (<0.001)	−0.704 (<0.001)	−0.810 (<0.001)	−0.831 (<0.001)	−0.805 (<0.001)	−0.730 (<0.001)
**NUTRITIONAL**	**0.820** (0.569)	0.144 (0.318)	0.010 (0.947)	0.169 (0.240)	0.058 (0.690)	0.000 (1.000)	−0.111 (0.444)	0.029 (0.840)	0.050 (0.731)	0.011 (0.938)
**COGNITIVE**	−0.614 (<0.001)	−0.327 (0.021)	−0.391 (0.005)	−0.457 (<0.001)	−0.368 (0.009)	−0.473 (<0.001)	−0.415 (0.003)	−0.487 (<0.001)	−0.399 (0.004)	−0.361 (0.010)
**EMOTIONAL**	−0.203 (0.158)	0.020 (0.892)	−0.277 (0.052)	−0.156 (0.278)	−0.233 (0.104)	−0.008 (0.958)	−0.239 (0.094)	−0.132 (0.362)	−0.183 (0.203)	−0.103 (0.475)
**SOCIAL**	0.162 (0.261)	0.080 (0.580)	0.030 (0.835)	0.111 (0.369)	0.027 (0.853)	0.137 (0.342)	−0.030 (0.839)	0.086 (0.552)	0.002 (0.987)	0.044 (0.763)
**GERIATRIC** **SYNDROMES**	−0.451 (<0.001)	−0.465 (<0.001)	−0.509 (<0.001)	−0.494 (<0.001)	−0.256 (0.073)	−0.486 (<0.001)	−0.449 (<0.001)	−0.546 (<0.001)	−0.482 (<0.001)	−0.441 (<0.001)
**SEVERE** **SYMPTOMS**	−0.004 (0.979)	0.051 (0.724)	0.115 (0.427)	0.088 (0.544)	−0.047 (0.748)	0.154 (0.285)	−0.021 (0.883)	0.106 (0.465)	0.173 (0.230)	−0.007 (0.961)
**DISEASES**	−0.055 (0.703)	−.210 (0.144)	−0.169 (0.239)	−0.047 (0.745)	−0.295 (0.038)	−0.259 80.070)	−0.202 (0.160)	−0.268 (0.060)	−0.181 (0.209	−0.356 (0.011)

The Spearman correlation coefficient (r) and the *p*-value were applied. In all analyses, *p* < 0.05 was considered statistically significant with a 95% confidence interval.

**Table 5:** Result of the Frail-VIG index (n (%)).

Frail-VIG Index						
Domain	Item	YES		NO		
**FUNCTIONAL**	**Item 1. Money management**	32 (64%)		18 (36%)		
	**Item 2. Using the telephone**	22 (44%)		28 (56%)		
	**Item 3. Medication control**	26 (52%)		24 (48%)		
**DEPENDENCE**	**Item 4. Barthel Index**	Without dependence (BI ≥ 95)		Mild–moderate dependence (BI 90–65)	Moderate–severe dependence (BI 60–25)	Absolute dependence (BI ≤ 20)
		6 (12%)		18 (36%)	16 (32%)	10 (20%)
**NUTRITIONAL**	**Item 5. Malnutrition**	YES	NO			
		34 (68%)	16 (32%)			
**COGNITIVE**	**Item 6. Degree of cognitive impairment**		No cognitive impairment	Mild–moderate	Serious, very serious	
			YES	16 (32%)	4 (8%)	
				30 (60%)	NO	
**EMOTIONAL**	**Item 7. Depressive syndrome**		10 (20%)	40 (80%)		
	**Item 8. Insomnia/Anxiety**		31 (62%)	19 (38%)		
**SOCIAL**	**Item 9. Social vulnerability**		5 (10%)	45 (90%)		
**GERIATRIC SYNDROMES**	**Item 10. Delirium**		10 (20%)	40 (80%)		
	**Item 11. Falls**		8 (16%)	42 (84%)		
	**Item 12. Ulcers**		14 (28%)	36 (72%)		
	**Item 13. Polypharmacy**		42 (84%)	8 (16%)		
	**Item 14. Dysphagia**		7 (14%)	43 (86%)		
**SEVERE** **SYMPTOMS**	**Item 15. Pain**		32 (64%)	18 (36%)		
	**Item 16. Dyspnea**		13 (26%)	37 (74%)		
**DISEASES**	**Item 17. Cancer**		50 (100%)	0 (0%)		
	**Item 18. Respiratory**		15 (30%)	35 (70%)		
	**Item 19. Cardiac**		21 (42%)	29 (58%)		
	**Item 20. Neurological**		7 (14%)	43 (86%)		
	**Item 21. Digestive**		17 (34%)	33 (66%)		
	**Item 22. Renal**		16 (32%)	34 (68%)		

VIG: Comprehensive Geriatric Assessment; BI: Barthel Index.

## Data Availability

The data that support the findings of this study are available from the corresponding author upon reasonable request. The data presented in this study are available on request from the corresponding author (E.N.-F.) upon reasonable request. The data statements are available at the following link http://consigna.uv.es/g?recoge:es:320_3391_1749_3885 (accessed on 29 November 2021).
